# Role of Starch Type in Gel-like Network Formation of Extruded Meat Analogs

**DOI:** 10.3390/gels12010094

**Published:** 2026-01-22

**Authors:** Chaeyeon Kang, Ayeon Han, Bon-Jae Gu

**Affiliations:** Department of Food Science and Technology, Food and Feed Extrusion Research Center, Kongju National University, Yesan 32439, Republic of Korea

**Keywords:** meat analogs, low- and high-moisture extrusion, gel-like network, starch–protein interactions, structure–property relationship

## Abstract

Starches play a crucial role in determining the expansion, texture, and structural development of extruded meat analogs through their gelatinization behavior and interactions with proteins. In this study, corn, pea, tapioca, sweet potato, and potato starches were incorporated into soy protein-based formulations and processed under low-moisture and high-moisture extrusion conditions to investigate starch-dependent physicochemical properties. Amylose/amylopectin composition and starch pasting properties were evaluated, and the resulting extrudates were characterized in terms of expansion behavior, water-related properties, textural attributes, and internal structure. Distinct differences in pasting behavior were observed among starches, with potato starch exhibiting high peak viscosity and pea starch showing strong viscosity development during cooling. These differences were closely associated with extrusion outcomes, influencing expansion ratio and texture formation. In low-moisture extrusion, starches susceptible to thermal and shear degradation showed increased solubilization, whereas in high-moisture extrusion, enhanced starch gelatinization promoted starch–protein interactions and contributed to improved textural integrity and structural alignment. Overall, the results demonstrate that starch type is a key determinant of expansion behavior, texture, and structural organization in extruded meat analogs, highlighting the importance of starch selection and processing conditions for tailoring product quality.

## 1. Introduction

By 2050, the global population is projected to reach approximately 9.8 billion, which is expected to drive a nearly 70% increase in agricultural demand [1,2]. This rapid population growth underscores the need for sustainable alternative food sources. Among these, meat analogs have gained increasing attention as a viable approach to reducing environmental burdens, improving animal welfare, and diversifying dietary options [3]. Accordingly, the global market for meat analogs has expanded rapidly and continues to attract significant interest [4]. Plant-based meat analogs are formulated using plant proteins, starches, lipids, and food-grade gums, and their meat-like texture and structure are achieved through physicochemical transformations induced during processing [5,6]. Several structuring technologies, including cultured meat, shear-cell processing, and extrusion, have been explored, with extrusion being the most widely adopted approach [7,8]. During extrusion, proteins and starches are subjected to high temperature, pressure, and shear, leading to physicochemical changes that promote fibrous structure formation and meat-like textures. This process offers advantages such as high productivity, cost efficiency, and flexibility in raw material selection [9,10]. Based on moisture content, extrusion can be classified into low-moisture and high-moisture processes. Plant-based meat analogs aim to mimic ground meat, minced meat, and whole cuts to achieve a texture similar to meat. They particularly strive to replicate the taste and texture of meat. Plant-based extruded meat analogs are classified into low-moisture and high-moisture processes based on their moisture content. Low-moisture extrusion (moisture content 15–40%) offers easy handling and storage but typically requires a rehydration process before consumption; when moisture is added, it exhibits a fibrous structure resembling meat. High-moisture extrusion (moisture content > 40%) produces products with a fibrous structure and juiciness similar to conventional meat, eliminating the need for a rehydration process [11].

Under high-moisture conditions, interactions between proteins and starches become more pronounced, promoting the formation of protein–starch complexes that enhance elasticity and chewiness in the final product [12]. From a structural perspective, these interactions can be interpreted as the formation of a continuous, water-mediated gel-like network that governs mechanical integrity and textural anisotropy. Such interactions critically influence the textural and physicochemical properties of meat analogs, with both starch selection and processing conditions serving as key determinants of product quality [12,13]. Starches are composed of amylose and amylopectin, which differ in molecular architecture, with amylose exhibiting a linear structure and amylopectin a highly branched configuration. These structural features govern functional properties such as gelatinization, swelling, and water absorption [14]. These molecular features also influence the ability of starch to contribute to continuous network formation and to interact with proteins under thermal and shear conditions.

Starches from different botanical sources, including corn, potato, tapioca, sweet potato, and pea, impart distinct functionalities to meat analog formulations. Corn starch is widely used due to its swelling capacity and economic advantages [15], whereas pea starch is valued for its availability and cost-effectiveness despite relatively low shear stability and acid resistance [16]. Tapioca starch provides high viscosity and clarity, contributing to desirable textural attributes [17]. Sweet potato starch exhibits high expansion capability even at low inclusion levels, while potato starch promotes fibrous structure formation owing to its excellent swelling behavior and viscosity [18]. Starch type plays a central role in the texturization of meat analogs. High-amylose starches have been reported to support structural development and enhance fibrousness and chewiness [19]. In high-moisture extrusion, pigeon pea starch has been shown to facilitate fibrous structure formation by increasing swelling and viscosity [12]. Moreover, interactions between amylose, amylopectin, and proteins can result in layered or aligned structures, with amylopectin contributing to protein realignment and fiber strengthening [20]. Collectively, these findings indicate that starch characteristics substantially influence the physicochemical properties and quality of extruded meat analogs. Accordingly, the present study investigates the role of starch type in gel-like network formation and physicochemical properties of extruded meat analogs.

## 2. Results and Discussion

### 2.1. Raw Material

#### 2.1.1. Determination of Amylose Content

**Figure 1:**
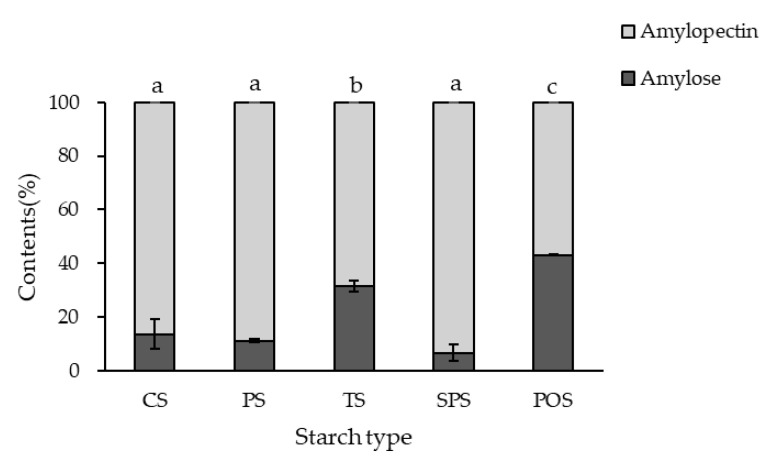
Amylose and amylopectin content in raw materials used for extruded meat analogs. CS, corn starch; PS, pea starch; TS, tapioca starch; SPS, sweet potato starch; POS, potato starch. Significant differences among groups (*p* < 0.05) were assessed by Tukey’s HSD test and are indicated by different letters.

The ratio of amylose to amylopectin is a critical factor governing starch viscosity, gelatinization behavior, swelling capacity, and gel-forming ability; therefore, the amylose and amylopectin contents of the starches were determined [21,22,23,24]. The amylose and amylopectin compositions of the raw starches used in this study are presented in Figure 1. Among the tested starches, potato starch (POS) exhibited the highest amylose content (43.18 ± 0.14%), whereas sweet potato starch (SPS) showed the lowest value (6.58 ± 3.07%). Amylose-rich starches are generally associated with the formation of stronger and more rigid gel structures due to enhanced chain–chain associations during gelatinization and cooling, whereas amylopectin-rich starches tend to form softer, more extensible gel-like matrices characterized by greater swelling and water retention. Accordingly, the large variation in amylose content observed among the starches suggests that their contributions to gel-like network formation during extrusion are likely to differ substantially. It should be noted that the measured amylose contents, particularly for potato and sweet potato starches, may be influenced by starch molecular architecture, including differences in amylopectin chain length and branching characteristics. The amylose content of potato starch measured in this study was found to be high, which may be attributed to the long external chains and spatially extended structure of potato starch amylopectin. Such structural characteristics could hinder complete precipitation of amylopectin by Con A, leaving some amylopectin fractions in the supernatant and thereby contributing to an overestimation of amylose content [25,26,27,28]. In addition, potato starch is known to have a relatively low inherent lipid content, allowing amylose to exist in a more freely dispersed state within the starch granule. Consequently, amylose may remain in the supernatant after Con A treatment, leading to an apparently elevated amylose value [29]. In contrast, the lower amylose content measured for sweet potato starch may be associated with its amylopectin structure, which is characterized by a shorter average chain length and the presence of finely branched chains. These structural features likely promote co-precipitation of amylopectin with Con A, thereby reducing the amount of amylose remaining in the supernatant and resulting in an underestimation of amylose content [30,31]. Such variation may also arise from differences in botanical origin, cultivar, environmental growth conditions, starch manufacturing processes, and post-manufacturing storage conditions, all of which can alter starch molecular structure and properties [32]. This variability in starch molecular composition is expected to influence gelatinization behavior under extrusion conditions and subsequent gel-like structural development. Previous studies have reported amylose contents of approximately 20–30% for corn starch, 17.2–19.2% for pea starch, 21–25% for tapioca starch, 17–24% for sweet potato starch, and 23.3–26.5% for potato starch [33,34]. The amylose/amylopectin ratios measured in the present study deviated from these reported ranges, which may be attributed to differences in botanical origin, cultivar, and environmental growth conditions [35,36].

#### 2.1.2. Effects of Starch Type on Pasting Properties

**Figure 2:**
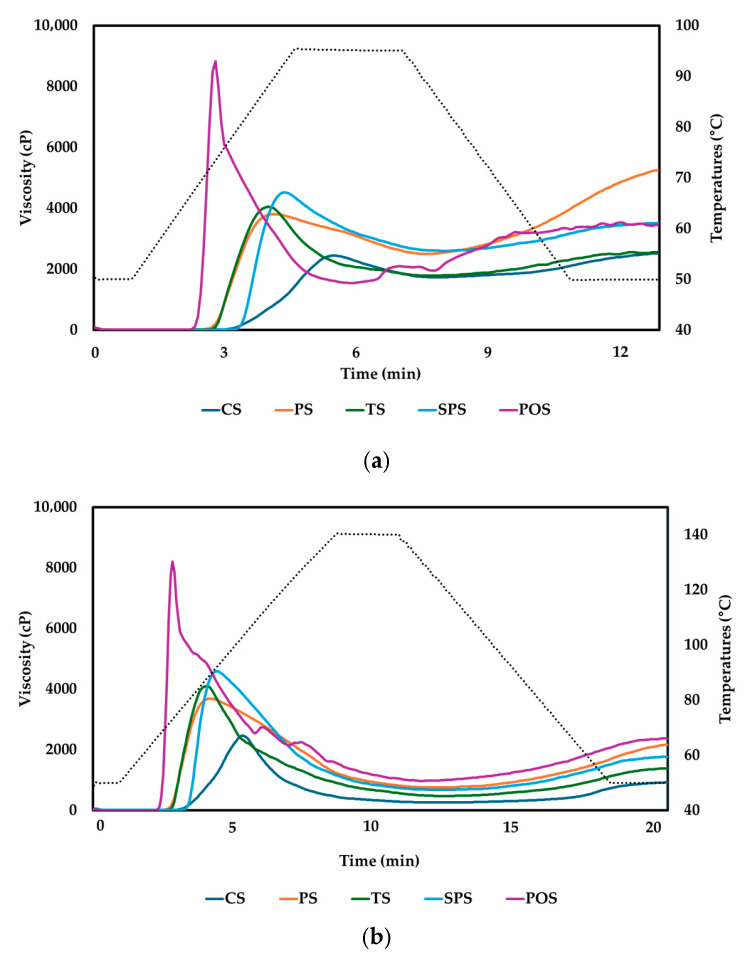
Pasting properties of starches determined by RVA at different target temperatures: (**a**) 95 °C profile; (**b**) 140 °C profile. CS, corn starch; PS, pea starch; TS, tapioca starch; SPS, sweet potato starch; POS, potato starch. The dotted line represents the temperature profile during the RVA measurement.

Under the 95 °C condition, potato starch (POS) exhibited the highest peak viscosity, followed by sweet potato starch (SPS), tapioca starch (TS), pea starch (PS), and corn starch (CS) in decreasing order (Figure 2a). In contrast, the highest final viscosity was observed for PS, followed by SPS, POS, TS, and CS. The markedly high peak viscosity and breakdown value of POS indicate rapid swelling and subsequent collapse of starch granules during heating. This behavior reflects the high swelling capacity and weak granular integrity of tuber starches, which are characterized by large granule size and low resistance to thermal and shear stress [37]. From a gel perspective, such extensive swelling followed by structural collapse suggests the formation of a transient gel-like structure with limited mechanical stability under shear. In contrast, PS exhibited a relatively lower peak viscosity but the highest final viscosity and setback among the tested starches. This behavior indicates pronounced starch chain reassociation during cooling, likely driven by enhanced intermolecular interactions formed during thermal treatment [38]. Such reassociation is indicative of the development of a more stable gel-like network, which may contribute to improved structural continuity during subsequent processing. CS showed the lowest peak viscosity and breakdown values under the 95 °C condition, suggesting greater granular integrity during heating. This stability is commonly attributed to amylose–lipid complex formation and stronger internal bonding within cereal starch granules, which restrict excessive swelling and reduce susceptibility to shear-induced disintegration [39]. Consequently, corn starch tends to form a more restrained but structurally robust gel matrix during pasting.

When the heating temperature was elevated to 140 °C, substantial changes in pasting behavior were observed for all starch types (Figure 2b). For most starches, peak viscosity did not increase significantly compared with the 95 °C condition, whereas holding strength and final viscosity decreased markedly. These changes indicate enhanced thermal- and shear-induced degradation of swollen starch granules and starch molecules at elevated temperatures. This behavior is consistent with previous high-temperature RVA studies reporting progressive viscosity loss at temperatures exceeding complete gelatinization due to granule disintegration and molecular breakdown [40]. Notably, starches with relatively higher amylose content and greater gelatinization resistance tended to retain higher final viscosity even under severe thermal conditions. This observation suggests that amylose-rich starches are more capable of maintaining gel-like network integrity at elevated temperatures, thereby promoting structural persistence under conditions relevant to extrusion processing. Similar trends have been reported in high-temperature RVA studies, where amylose-rich starches maintained higher final viscosity and gel strength compared with low-amylose starches under extreme thermal and shear environments [40,41]. In addition to viscosity magnitude, the time required to reach peak viscosity differed among starches but remained comparable between the 95 °C and 140 °C conditions. This indicates that increasing the heating temperature did not significantly affect the timing of peak viscosity development. Instead, peak time was primarily governed by intrinsic starch properties. Starches rich in amylopectin generally reach peak viscosity more rapidly due to faster water uptake and granule swelling, whereas increased amylose content restricts swelling and enhances granule stability, resulting in delayed peak viscosity development [42]. Although potato starch, sweet potato starch, and tapioca starch are all classified as tuber starches, their gelatinization and pasting behaviors differ markedly depending on starch type. These differences have been attributed to variations in granule size distribution, phosphate monoester content, and molecular structural characteristics. Potato starch exhibits pronounced swelling and higher viscosity due to its relatively high phosphate content and larger granule size, whereas sweet potato and tapioca starches show distinct breakdown behavior and thermal stability associated with differences in molecular structure and swelling resistance [43,44,45].

### 2.2. Effects of Starch Type on the Physicochemical Properties of Extruded Meat Analogs

#### 2.2.1. Effects of Starch Type on Expansion Behavior

Low- and high-moisture extrusion can be regarded as two structurally distinct extrusion systems characterized by different mechanisms of structure formation. Accordingly, the results presented in Section 2.2 are organized to outline the inherent structural features of each system, providing a reference framework for interpreting starch-type-dependent effects discussed in the following subsections. The expansion ratios of the low-moisture extruded meat analogs (LMMA) are presented in Table 1. Significant differences were observed among samples depending on the type of starch incorporated. TS exhibited the lowest expansion ratio (2.92 ± 0.12), whereas POS showed the highest value (6.00 ± 0.53). Expansion behavior in low-moisture extrusion is closely associated with starch gelatinization, melt elasticity, and the ability to form and stabilize a gel-like matrix during pressure release. The amylose-to-amylopectin ratio has been reported as a key factor governing expansion characteristics [46]. According to previous studies, the amylose content of corn, pea, tapioca, sweet potato, and potato starches ranges from 20–30%, 17.2–19.2%, 21–25%, 17–24%, and 23.53–26.5%, respectively [33,34]. In general, starches with higher amylose content tend to exhibit reduced expansion, as the predominantly linear structure of amylose restricts starch swelling and bubble growth during extrusion [46]. Consistent with this behavior, TS exhibited a relatively low expansion ratio, which can be attributed to limited swelling and reduced melt expansion.

Interestingly, despite its high amylose content, POS exhibited the greatest expansion ratio among the tested starches. This apparent contradiction may be explained by the combined effects of extensive granule swelling prior to collapse and the presence of resistant starch, which can interact with soy protein to form a viscoelastic, hydrocolloid-like gel matrix. Such interactions may enhance melt strength during pressure release, thereby promoting expansion while preventing premature collapse [47]. In this context, the expansion behavior of POS suggests the formation of a transient but sufficiently strong gel-like structure capable of sustaining expansion under low-moisture extrusion conditions. Amylopectin-rich starches generally facilitate expansion by promoting starch granule swelling and vapor bubble growth during extrusion. However, because amylopectin contributes limited network rigidity, excessive swelling may result in structural instability and collapse of the expanded matrix [48]. These results indicate that optimal expansion in low-moisture meat analogs is governed not solely by amylose content, but by the balance between swelling capacity, gel-like network strength, and melt stability during extrusion.

#### 2.2.2. Effects of Starch Type on Water Holding Capacity

Water holding capacity (WHC) reflects the ability of meat analogs to retain water after hydration and is closely related to internal structure and network formation [49]. The WHC values of both low-moisture and high-moisture extrudates are summarized in Table 1. Among the low-moisture samples, the ISP control exhibited the lowest WHC (4.08 ± 0.71 g/g). Incorporation of starch resulted in an overall increase in WHC, and although no significant differences were observed among the starch-containing samples, all showed significantly higher values than ISP. The enhanced WHC of starch-containing low-moisture extrudates can be attributed to the development of a porous structure formed by rapid pressure release at the die exit, which increases water uptake and retention capacity [50]. In addition, starch gelatinization during extrusion contributes to the formation of a continuous gel-like matrix that entraps water within the expanded structure. Notably, a positive relationship was observed between expansion ratio and WHC in low-moisture meat analogs, suggesting that highly expanded structures provide greater internal void volume and hydroxyl-rich surfaces capable of retaining water through intramolecular and intermolecular hydrogen bonding [51,52,53]. A similar trend was observed for high-moisture meat analogs, where ISP again exhibited the lowest WHC (1.16 ± 0.31 g/g). In contrast, CS and POS showed relatively higher WHC values (2.63 ± 0.58 g/g and 2.78 ± 0.35 g/g, respectively). Unlike low-moisture extrusion, high-moisture extrusion using a cooling die suppresses expansion and promotes the formation of a dense, layered fibrous structure, which enhances water retention by limiting moisture loss during processing [54]. Furthermore, interactions between starch and protein under high-moisture conditions facilitate the development of a gel-like network, increasing the water-binding capacity of the matrix despite the absence of macroscopic expansion [55]. However, the overall WHC of high-moisture meat analogs remained lower than that of low-moisture counterparts, which is likely associated with the formation of a compact, protein-dominated gel network with limited free volume available for macroscopic water entrapment.

#### 2.2.3. Water-Related Functional Indices

**Figure 3:**
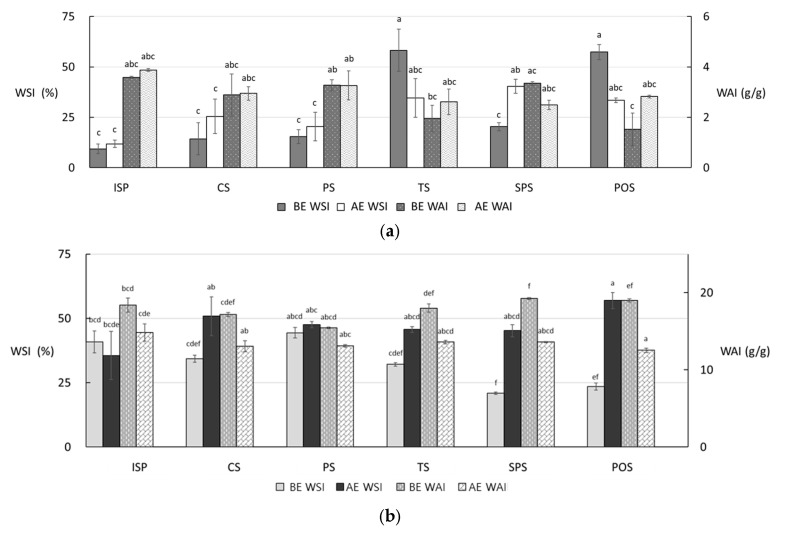
WSI and WAI of meat analogs produced with starches: (**a**) LMMA; (**b**) HMMA. LMMA, low-moisture meat analog; HMMA, high-moisture meat analog; BE, before extrusion; AE, after extrusion; ISP, isolated soy protein; CS, corn starch; PS, pea starch; TS, tapioca starch; SPS, sweet potato starch; POS, potato starch. Significant differences among groups (*p* < 0.05) were assessed by Tukey’s HSD test and are indicated by different letters.

The water solubility index (WSI) is commonly used to assess the extent of starch molecular degradation and solubilization, whereas the water absorption index (WAI) reflects the water-binding and swelling capacity of starch, which are closely related to gel formation and network integrity [56]. Changes in WSI and WAI before and after low- and high-moisture extrusion are presented in Figure 3. Among the low-moisture extrudates, sweet potato starch (SPS) exhibited the greatest increase in WSI, rising from 20.33 ± 2.01% to 40.32 ± 3.40%. This increase is likely attributable to extensive starch degradation during extrusion, resulting in the formation of soluble low-molecular-weight polysaccharides such as dextrins [57]. In contrast, the WSI values of tapioca starch (TS) and potato starch (POS) decreased substantially, from 58.24 ± 10.45% to 34.56 ± 9.58% and from 57.33 ± 3.80% to 33.45 ± 1.22%, respectively. These reductions suggest limited molecular solubilization, which may be associated with insufficient moisture availability or incomplete disruption of starch granules under low-moisture extrusion conditions [57,58]. From a structural perspective, reduced WSI in these samples implies the retention of larger starch fragments that can contribute to the formation of a more continuous gel-like matrix.

For high-moisture meat analogs, WSI increased markedly in samples containing corn starch (CS), SPS, and POS, increasing from 34.23 ± 1.37% to 50.83 ± 7.46%, from 20.89 ± 0.44% to 45.20 ± 2.31%, and from 23.48 ± 1.37% to 56.99 ± 3.12%, respectively. These increases indicate enhanced starch gelatinization and molecular dispersion under high-moisture conditions, which facilitate greater interaction between starch molecules and water [59]. Such behavior reflects partial disruption of the gel network, leading to the release of soluble starch fractions. In contrast, all samples exhibited a decrease in WAI following high-moisture extrusion. This trend can be attributed to the formation of a denser protein-dominated network under high moisture and temperature, which restricts starch swelling and limits water accessibility within the matrix [60]. Moreover, elevated moisture levels can suppress excessive starch swelling [61] and promote starch–protein complex formation, resulting in a more compact gel-like structure with reduced water absorption capacity [62].

Generally, an increase in WSI after low-moisture extrusion is attributed to the intensive thermal and shear-induced degradation of starch macromolecules, which reduces molecular weight and promotes the formation of soluble low-molecular-weight fractions [63]. However, the present results demonstrate that this trend is strongly dependent on starch type. Tapioca starch (TS) and potato starch (POS) exhibited a substantial decrease in WSI after low-moisture extrusion. This unexpected reduction is likely due to the large granule size and high swelling capacity of these tuber starches. Under limited moisture availability (LMMA), insufficient water restricts complete starch solubilization, resulting in the retention of swollen but structurally intact starch domains that entrap potentially soluble fractions, thereby lowering the measurable WSI despite high processing temperatures [64,65]. In contrast, WSI values were consistently higher in high-moisture meat analogs (HMMA) compared with their low-moisture counterparts. Although higher moisture content typically reduces overall shear intensity due to lower friction, high-moisture conditions facilitate extensive starch hydration and more complete gelatinization. Under such hydration-dominant environments, starch solubilization is governed primarily by gelatinization-driven molecular mobility and dispersion rather than by mechanical shear-induced fragmentation [63,66]. This explains the paradox of higher WSI under lower shear conditions, as the increased water availability allows starch chains to disentangle and disperse more effectively into the matrix. Comparing the two systems, the starch-specific behavior becomes even more evident. In HMMA, the increase in WSI was accompanied by a simultaneous decrease in WAI across all starch types. This inverse relationship suggests the formation of a dense and continuous protein-dominated gel-like network. In this compact structure, starch molecules are more thoroughly gelatinized (increasing WSI), yet their physical swelling and water uptake are mechanically restricted by the surrounding protein matrix (decreasing WAI). Similar WSI-WAI trade-off behavior has been interpreted as an indicator of network compactness and restricted swelling capacity [65,67]. Therefore, WSI and WAI must be interpreted in combination. The elevated WSI observed in HMMA does not contradict the reduced shear intensity but instead reflects a transition from shear-induced molecular degradation to hydration-driven network formation, in which starch acts as a functional filler contributing to structural integrity and water-related stability of the meat analog.

#### 2.2.4. Appearance

**Figure 4:**
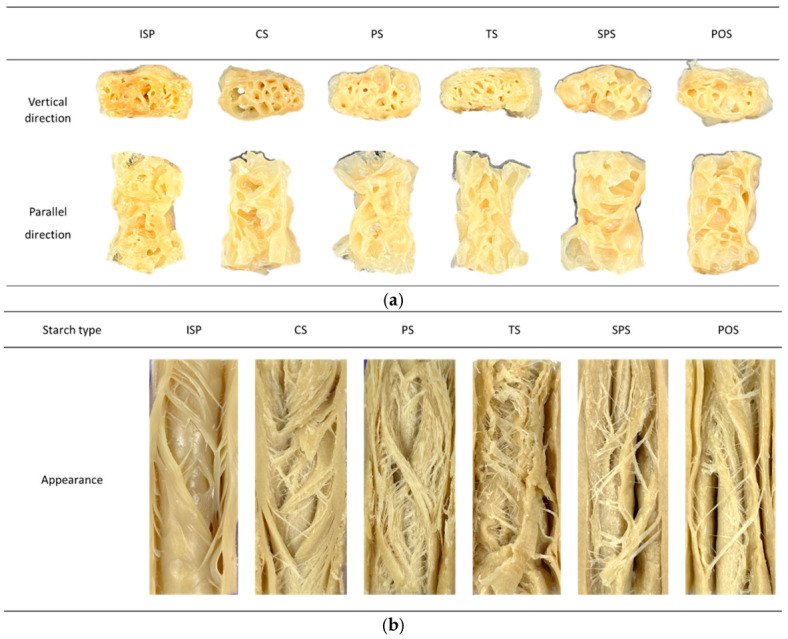
Appearance of extruded meat analogs produced with starches: (**a**) low-moisture extruded samples; (**b**) high-moisture extruded samples; ISP, isolated soy protein; CS, corn starch; PS, pea starch; TS, tapioca starch; SPS, sweet potato starch; POS, potato starch.

The appearance of the extruded meat analogs prepared with different starch types is shown in Figure 4. The low-moisture samples exhibited a porous internal structure, characterized by numerous air cells observed in both flow-vertical and flow-parallel cross-sections. This porous morphology can be attributed to the rapid pressure drop at the die exit, which induces expansion of entrapped vapor and air within the molten matrix [68]. From a structural standpoint, such expansion reflects the formation of a discontinuous, foam-like gel structure in which the gel matrix stabilizes gas cells generated during extrusion.

In contrast, the high-moisture meat analogs displayed more compact and well-aligned fibrous structures, particularly in samples containing corn starch (CS), pea starch (PS), and sweet potato starch (SPS). These starches are characterized by relatively high amylopectin content, which has been reported to facilitate molecular alignment and layer formation under shear-dominant extrusion conditions [69]. The suppression of expansion by the cooling die promotes the development of a dense, continuous gel-like network, resulting in anisotropic fibrous structures rather than porous morphologies. These observations suggest that starch composition influences not only macroscopic appearance but also the underlying gel-like structural organization formed during extrusion.

#### 2.2.5. Texture Properties

The textural properties of the extruded meat analogs containing different starches are summarized in Table 2. For the hydrated low-moisture samples, sweet potato starch (SPS) exhibited the lowest springiness (84.60 ± 3.59%), while corn starch (CS) and pea starch (PS) showed relatively low chewiness values (378.21 ± 110.05 g and 523.41 ± 164.52 g, respectively). SPS also demonstrated the lowest cohesiveness (57.34 ± 10.70%). These reductions in elastic and cohesive responses indicate that the incorporation of certain starches weakens the continuity of the gel-like matrix formed under low-moisture extrusion conditions. In the high-moisture meat analogs, tapioca starch (TS) and SPS exhibited reduced springiness (73.58 ± 2.44%, 67.66 ± 0.04%, and 63.87 ± 0.07%, respectively), accompanied by lower chewiness values (TS: 446.71 ± 234.43 g; SPS: 369.29 ± 117.49 g). Cohesiveness was lowest in TS (41.82 ± 0.03%). Overall, starch incorporation tended to reduce springiness, chewiness, and cohesiveness in both extrusion systems, consistent with previous reports that starch addition softens meat analog textures by disrupting or diluting the load-bearing protein-dominated gel network [70]. From a structural perspective, starch granules and gelatinized starch domains can act as discontinuities within the gel matrix, thereby reducing elastic recovery and internal cohesion.

The incorporation of starch tended to reduce cutting strength in both low- and high-moisture meat analogs. For low-moisture meat analogs, flow-vertical cutting strength decreased for all treatments except TS, with CS exhibiting the lowest value (136.23 ± 23.31 g/cm^2^). Flow-parallel cutting strength showed a similar decreasing trend, again with CS presenting the lowest value (96.15 ± 28.96 g/cm^2^). Comparable patterns were observed in the high-moisture samples, where cutting strength in both directions decreased for all starch-containing treatments except TS, and CS recorded the lowest values in both the vertical and parallel directions (136.23 ± 23.31 g/cm^2^ and 96.15 ± 28.96 g/cm^2^, respectively). The consistently low cutting strength observed for corn starch (CS) is considered to result from its limited contribution to the formation of a continuous starch–protein network during extrusion, leading to a porous but mechanically weak structure in both low- and high-moisture meat analogs. In contrast, the exceptional cutting strength of tapioca starch (TS) is likely attributable to its suppressed expansion, partial starch solubilization, and delayed setback behavior, which are presumed to facilitate effective shear-induced alignment and enhanced textural organization under low-moisture extrusion conditions, as previously reported for starches exhibiting amylopectin-dominant pasting behavior under high thermal and shear processing [40].

The reduction in cutting strength reflects a decrease in the mechanical resistance of the gel-like network, indicating that starch incorporation modifies not only bulk texture but also directional structural integrity. Differences between flow-vertical and flow-parallel cutting strengths suggest that starch type influences the degree of anisotropy in the gel network formed during extrusion. Collectively, these results demonstrate that starch type plays a critical role in modulating gel network strength, continuity, and mechanical anisotropy, thereby enabling targeted control of textural properties in extruded meat analogs. The role of starch in network formation depended strongly on the moisture-regulated structural state of soy protein during extrusion. Under low-moisture conditions, limited water availability promotes rapid protein denaturation and aggregation, resulting in discontinuous and porous structures dominated by expansion. In contrast, high-moisture extrusion facilitates protein unfolding and molecular rearrangement, enabling the formation of continuous gel-like networks. Within this hydrated protein matrix, gelatinized starch acts as a structural filler and interaction mediator, thereby enhancing network continuity and integrity. Overall, these results suggest that the effects of starch on texture and structure are primarily governed by moisture-dependent protein structural transitions during extrusion, rather than by starch properties alone.

#### 2.2.6. Structural Texturization and Integrity Characteristics

The degree of texturization and integrity index of the extruded meat analogs are presented in Table 3. The degree of texturization reflects the extent of fibrous structural development in hydrated meat analogs and is commonly used as an indicator of directional alignment and textural anisotropy [71]. Among the low-moisture samples, tapioca starch (TS) exhibited the highest degree of texturization (2.42 ± 0.22), indicating enhanced fiber alignment under low-moisture extrusion conditions. This suggests that TS promoted shear-induced orientation of the matrix, contributing to the development of a more pronounced fibrous structure. This behavior can be attributed to the physicochemical characteristics of TS observed in this study. Compared with potato and pea starches, TS exhibited moderate pasting viscosity and a relatively stable paste structure, avoiding excessive granule collapse or rigid gel formation. In addition, the amylopectin-rich composition of TS likely facilitated the formation of a cohesive yet deformable matrix under limited moisture availability, enabling effective flow-induced alignment during high-shear extrusion. The reduced WSI of TS under low-moisture conditions further suggests retention of starch domains within the matrix, which may have contributed to maintaining structural continuity and enhancing fiber orientation. By comparison, no significant differences were observed among the high-moisture samples, implying that starch type had a limited influence on the degree of texturization under conditions where fiber formation is predominantly governed by protein alignment within a continuous gel-like network.

The integrity index represents the ability of the extrudate to maintain structural cohesion and physical stability after hydration and mechanical stress. In the low-moisture samples, no significant differences in integrity index were observed among treatments. This indicates that, despite increased texturization in some formulations, the gel-like network formed under low-moisture conditions lacked sufficient continuity and strength to preserve overall structural integrity, likely resulting in partial collapse of the porous matrix after processing [48]. These results suggest that starch incorporation alone is insufficient to reinforce the load-bearing network in low-moisture meat analogs. In contrast, the integrity index of high-moisture meat analogs increased notably in samples containing corn starch (CS), pea starch (PS), and potato starch (POS), with values of 44.63 ± 4.84%, 44.86 ± 5.36%, and 40.91 ± 6.51%, respectively. The higher integrity index observed in these samples indicates improved physical stability and resistance to structural disruption, consistent with the formation of a dense and continuous gel-like network under high-moisture extrusion conditions. Similar structure–property relationships have been reported in multicomponent biopolymer systems, where enhanced intermolecular interactions promote network densification and improved mechanical integrity. In this context, the starch-dependent enhancement in integrity index observed under high-moisture extrusion can be attributed to strengthened starch–protein interactions governing gel-like network continuity [72]. Notably, TS and sweet potato starch (SPS) showed no significant improvement in integrity index (21.75 ± 4.60% and 5.15 ± 3.15%, respectively), suggesting limited contributions to network reinforcement under high-moisture conditions. Collectively, these results demonstrate that the effectiveness of starch addition in enhancing fibrous structure and structural stability is strongly dependent on moisture conditions. In low-moisture systems, starches may facilitate fiber orientation but do not substantially reinforce gel network continuity or mechanical integrity. In high-moisture extrusion, however, selected starches, particularly CS, PS, and POS, contribute more effectively to the stabilization of gel-like fibrous networks. These findings highlight the importance of selecting starch types in accordance with moisture conditions to tailor the structural performance and textural stability of extruded meat analogs.

## 3. Conclusions

This study investigated the effects of starch type on the physicochemical properties and structural development of low-moisture and high-moisture extruded meat analogs. Differences in amylose and amylopectin content among starches demonstrated their distinct roles in governing starch gelatinization, pasting behavior, and gel-like network formation during extrusion. Potato starch exhibited a relatively high amylose proportion, whereas sweet potato starch showed a lower amylose content, reflecting inherent molecular differences among botanical sources. Variations in amylose/amylopectin ratios compared with previously reported values were attributed to differences in cultivar and growth conditions. RVA analysis revealed starch-dependent differences in pasting behavior, with potato starch showing high peak viscosity at 95 °C due to extensive swelling, and pea starch exhibiting high final viscosity associated with starch chain reassociation during cooling. At elevated temperature (140 °C), the pronounced reduction in holding strength and final viscosity across starches indicated thermal- and shear-induced molecular degradation, highlighting differences in gel stability under severe processing conditions. Water-related properties further reflected moisture-dependent structural organization. In low-moisture extrusion, changes in WSI and expansion behavior were associated with starch degradation and the formation of porous, discontinuous gel structures. In high-moisture extrusion, increased WSI and reduced WAI indicated enhanced starch gelatinization, starch–protein interactions, and the formation of dense, continuous gel-like networks that governed water retention and structural stability. Multiple structural and mechanical indicators consistently showed that starch incorporation modulated gel network continuity, strength, and anisotropy in a moisture-dependent manner. In low-moisture systems, starch addition facilitated fiber orientation but was insufficient to reinforce overall network integrity. In contrast, selected starches in high-moisture extrusion, particularly corn, pea, and potato starches, contributed to the stabilization of fibrous gel-like structures with improved physical integrity. Overall, these findings confirm that starch type plays a critical role in regulating gel-like network formation, structural stability, and textural performance in extruded meat analogs. Appropriate selection of starch type, in combination with optimized moisture conditions, provides an effective strategy for tailoring the structural and functional properties of meat analog products.

Although the present study did not include direct molecular or rheological analyses, gel-like network formation was consistently reflected across multiple macroscopic indicators, including water-related properties, textural parameters, cutting strength, and structural indices, which captured moisture-dependent differences in network continuity and anisotropic structure development induced by starch type. The agreement between starch pasting behavior and extrusion outcomes supports the role of starch gelatinization and starch–protein interactions in governing gel network formation; nevertheless, direct analytical techniques such as oscillatory rheology or high-resolution microscopy would be valuable to further elucidate the underlying molecular mechanisms and should be addressed in future studies. In this context, although sensory evaluation was not conducted, the instrumental textural and structural parameters measured provide meaningful insight into expected consumer-relevant attributes. Higher cutting strength and integrity index are generally associated with increased firmness and chewiness, whereas pronounced anisotropy is indicative of a fibrous mouthfeel, and enhanced network continuity and water-holding capacity are commonly linked to improved juiciness perception. Accordingly, the starch-dependent differences observed in mechanical and structural properties are expected to translate into distinct sensory profiles, highlighting starch selection as an effective strategy for tailoring consumer-perceived texture in extruded meat analogs.

It should be noted that the starch effects identified in this study were evaluated at a fixed ISP/starch ratio of 80:20. Variations in starch concentration are expected to shift the balance between protein-dominated and starch-dominated gel networks, leading to nonlinear changes in expansion behavior under low-moisture conditions and concentration-dependent stabilization or disruption of fibrous structures under high-moisture extrusion. Accordingly, starch concentration should be considered alongside starch type and moisture level when interpreting and extending the present findings.

## 4. Materials and Methods

### 4.1. Experimental Materials

The experimental formulations employed isolated soy protein (ISP) as the protein matrix, which was supplied by Pingdingshan TianJing Plant Albumen Co., Ltd. (Henan, China). Five starches, corn starch (Samyang Co., Ltd., Ulsan, Republic of Korea), pea starch (Ingredion Co., Ltd., Westchester, NY, USA), and tapioca, sweet potato, and potato starches (Young Heung Groceries Co., Ltd., Eumseong-gun, Republic of Korea), were incorporated as functional carbohydrate components.

### 4.2. Extrusion Process

**Figure 5:**
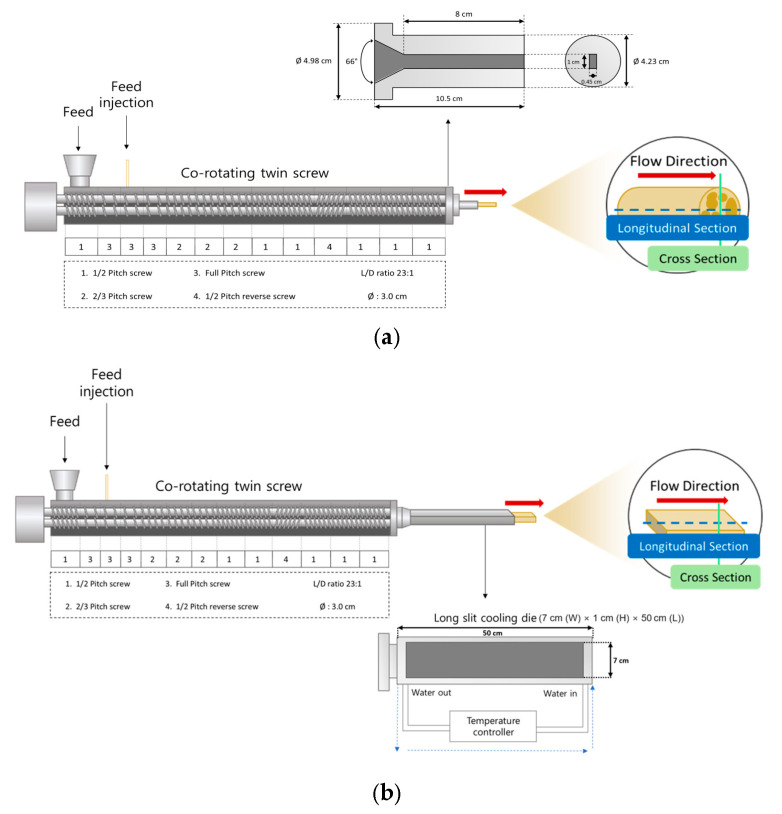
Schematic representation of the extrusion processes: (**a**) low-moisture extrusion; (**b**) high-moisture extrusion. The red arrows indicate the material flow direction during extrusion. The horizontal blue dashed line represents the longitudinal section, while the vertical green solid line denotes the cross section of the extrudates.

The production of low- and high-moisture meat analogs was carried out using a co-rotating twin-screw extruder (THK31T, Incheon Machinery Co., Ltd., Incheon, Republic of Korea) (Figure 5). Two distinct moisture regimes were applied, with moisture levels of 35% and 60% corresponding to LMMA and HMMA, respectively. Low- and high-moisture extrusion were treated as two distinct extrusion systems in this study. High-moisture extrusion inherently requires a cooling die to stabilize the aligned protein matrix under high-moisture conditions, whereas low-moisture extrusion relies on expansion and moisture flash-off at the die exit. Accordingly, extrusion operating conditions and formulation parameters were maintained constant within each extrusion system, including a barrel temperature of 150 °C and a screw speed of 200 rpm. Barrel temperatures were controlled using an electric heater in combination with a cooling-water circulation system. The formulations consisted of isolated soy protein (80%, *w*/*w*) and starch (20%, *w*/*w*), with corn starch (CS), pea starch (PS), tapioca starch (TS), sweet potato starch (SPS), or potato starch (POS) used as the starch source. For low-moisture extrusion, the extrudates were cut into uniform pieces and dried in a hot-air dryer (FC-PO-250, Lap House, Pcheon, Republic of Korea) at 50 °C for 10 h, followed by storage in plastic zipper bags. High-moisture extrudates were stored at 4 °C in a laboratory refrigerator (FSR2-650Z, Jeio Tech Co., Ltd., Daejeon, Republic of Korea). For physical property analysis, both low-moisture and high-moisture meat analogs were prepared as 1 cm × 1 cm cubes and analyzed for water-holding capacity, expansion ratio, texture profile, and degree of texturization. For chemical analyses, low-moisture samples were dried using hot-air drying, whereas high-moisture samples were freeze-dried (HyperCOOL 3110, LABOGENE Co., Ltd., Gimpo, Republic of Korea). All dried samples were subsequently ground and sieved to obtain powders with particle sizes ranging from 50 to 70 mesh (US Standard Sieve Series).

### 4.3. Raw Material Analysis

#### 4.3.1. Determination of Amylose Content in Starch

Amylose content was determined using a commercial Amylose/Amylopectin Assay Kit (K-AMYL, Megazyme International Ireland Ltd., Wicklow, Ireland) according to the manufacturer’s instructions. The amylose content was quantified by measuring absorbance at 510 nm using a UV-Vis spectrophotometer.

#### 4.3.2. Starch Pasting Properties

The pasting properties of starch samples were analyzed using a Rapid Visco Analyzer (RVA 4800; Perten Instruments, PerkinElmer, Sydney, NSW, Australia) following AACC Method 76-21.01 [73], with slight modifications based on a previously reported high-temperature RVA procedure [40]. All starch samples were prepared on a 14% moisture basis. For each measurement, approximately 3.0 g of starch (as-is basis) was mixed with 25.0 ± 0.1 mL of distilled water to obtain a starch suspension. Two temperature profiles were applied to investigate starch pasting behavior: a conventional profile with a maximum temperature of 95 °C and a high-temperature profile with a maximum temperature of 140 °C. For the 140 °C measurement, a sealed high-temperature canister (pressure vessel) of the RVA 4800 was used, allowing the system to self-pressurize during heating and thereby prevent boiling and evaporation of water above 100 °C. In contrast, the 95 °C measurement was conducted under conventional RVA conditions. The suspension was equilibrated at 50 °C for 1 min, heated to the target temperature at a rate of 12 °C/min, held for 2.5 min, cooled to 50 °C at the same rate, and maintained at 50 °C for an additional 2 min. The paddle speed was set to 960 rpm for the initial 10 s to ensure complete dispersion and subsequently maintained at 160 rpm for the remainder of the test. Viscosity data were recorded at 4-s intervals. All measurements were conducted in triplicate.

### 4.4. Analysis of the Physicochemical Properties of Extruded Meat Analogs

#### 4.4.1. Expansion Ratio

The expansion ratio of the low-moisture extrudates was determined by measuring the cross-sectional width and height at ten randomly selected positions using a digital vernier caliper (FUTURO IP67 Connected, Brutsch/Ruegger Tools GmbH, Swithland, Germany). The expansion ratio was calculated as the ratio of the die cross-sectional area to that of the extrudate, averaged over all measurements [74].

#### 4.4.2. Water Solubility Index (WSI) and Water Absorption Index (WAI)

Water-related functional properties were evaluated using a modified method described previously [75]. Briefly, 0.5 g of sample (dry basis) was mixed with 6 mL of distilled water and centrifuged at 2700× *g* for 15 min. The supernatant was carefully transferred to an aluminum weighing dish and dried in a hot-air oven at 105 °C to determine the soluble solid content. The centrifuge tube containing the sediment was weighed to determine water absorption. WSI and WAI values were obtained according to Equations (1) and (2), respectively.

(1) $$\mathrm{WAI}\;(g/g)=\;\frac{\mathrm{Sediment}\;\mathrm{wt}.}{\mathrm{Dry}\;\mathrm{solid}\;\mathrm{wt}.}\;$$

(2) $$WSI\;(\%)=\;\frac{\mathrm{Dissolved}\;\mathrm{solid}\;\mathrm{in}\;\mathrm{supernatant}\;\mathrm{wt}.}{\mathrm{Dry}\;\mathrm{sample}\;\mathrm{wt}.}\;\times \;100$$

#### 4.4.3. Water Holding Capacity (WHC)

The water holding capacity (WHC) of the extruded meat analogs was determined using a modified method based on a previously reported procedure [76]. Samples (approximately 1.5–2.0 cm in size) were immersed in a water bath at 90 °C for 20 min to allow complete hydration. After hydration, excess surface water was removed by draining the samples through a 20-mesh sieve for 15 min. The WHC was calculated according to Equation (3).

(3) $$\mathrm{WHC}\;(g/g)=\;\frac{\mathrm{Mass}\;\mathrm{after}\;\mathrm{hydration}-\mathrm{Dry}\;\mathrm{mass}}{\mathrm{Dry}\;\mathrm{mass}}$$

#### 4.4.4. Texture Analysis

The textural properties of the meat analogs were evaluated based on a modified method adapted from a previously reported study on extruded meat analogs [76]. The textural properties of the meat analogs were evaluated according to moisture condition. Prior to analysis, low-moisture samples underwent hydration in a water bath maintained at 90 °C for 20 min, whereas high-moisture samples were analyzed without any pretreatment. Texture measurements were performed by a texture analyzer (ZwickiLine Z0.5 TS, Zwick Roell, Germany). Compression tests were conducted using a cylindrical probe (Ø 100 mm) to determine elasticity, cohesiveness, and chewiness. Cutting strength was measured using a Warner-Bratzler shear device equipped with a cutting blade (0.75 cm × 3.83 cm). Each extrudate was cut either perpendicular to the extrusion direction (flow-vertical, FV) or parallel to the extrusion direction (flow-parallel, FP) to evaluate structural anisotropy. Elasticity, cohesiveness, chewiness, and cutting strength were calculated using Equations (4)–(7), respectively. The degree of texturization was determined based on the ratio of FV to FP values [77] and calculated according to Equation (8). All measurements were conducted in six replicates.

(4) $$\mathrm{Springiness}\;(\%)=\frac{D_{2}}{D_{1}}\;\times \;100$$

*D*_1_: Distance when first occurred maximum stress; *D*_2_: Distance when second occurred maximum stress.

(5) $$\mathrm{Cohesiveness}\;(\%)=\frac{A_{2}}{A_{1}}\;\times \;100$$

A_1_: Area when first occurred maximum stress; A_2_: Area when second occurred maximum stress.

(6) $$\mathrm{Chewiness}\;(g)=\;\frac{\mathrm{Springiness}}{100}\times \frac{\mathrm{Cohesiveness}}{100}\times Maximum\;stress\;$$

(7) $$\mathrm{Cutting}\;\mathrm{strength}\;(g/cm^{2})=\;\frac{\mathrm{Maximum}\;\mathrm{stress}}{\mathrm{Cross}\;\mathrm{sectional}\;\mathrm{area}}$$

(8) $$\mathrm{Degree}\;\mathrm{of}\;\mathrm{texturization}=\;\frac{F_{V}}{F_{P}}$$

F_V_: Flow Direction Vertical; F_P_: Flow Direction Parallel.

#### 4.4.5. Integrity Index

The integrity index of the extruded meat analogs was determined to evaluate structural stability under thermally and mechanically demanding conditions, following a modified method based on a previously reported procedure [78]. Low-moisture samples were hydrated in a water bath at 90 °C for 20 min prior to analysis, whereas high-moisture samples were analyzed without pretreatment. Approximately 3 g of sample was mixed with 100 mL of distilled water and subjected to thermal treatment at 121 °C for 15 min under pressurized conditions. After then, the samples were cooled under tap water for 30 s. Following hydration, homogenization was performed at 5000 rpm for 30 s in 100 mL of distilled water using a homogenizer (HG-15D homogenizer, DAIHAN Scientific Co., Ltd., Wonju, Republic of Korea). The resulting homogenate was passed through a 20-mesh sieve to separate insoluble residues. The collected residues were dried in a hot-air oven at 105 °C for 8 h. The integrity index was calculated based on the ratio of the dried residue weight to the initial sample weight using Equation (9).

(9) $$\mathrm{Integrity}\;\mathrm{index}\;(\%)=\frac{\mathrm{Dry}\;\mathrm{residue}\;\mathrm{wt}.}{\mathrm{Sample}\;\mathrm{wt}.}\;\times \;100$$

#### 4.4.6. Statistical Analysis

Statistical analyses were performed using SPSS software (Version 23.0; IBM-SPSS, Thornwood, NY, USA). Data were analyzed by one-way analysis of variance (ANOVA), and when significant differences were detected, mean comparisons were conducted using Tukey’s honestly significant difference (HSD) test at a significance level of *p* < 0.05.

## Figures and Tables

**Table 1:** Expansion ratio and water holding capacity (WHC) of low- and high-moisture extruded meat analogs with different starch types.

	Starch Types	Expansion Ratio	WHC (g/g)
LMMA ^(1)^	ISP ^(2)^	3.47 ± 0.22 ^d(4)^	4.08 ± 0.71 ^b^
	CS	4.70 ± 0.34 ^c^	5.81 ± 0.59 ^a^
	PS	5.39 ± 0.53 ^b^	6.25 ± 0.45 ^a^
	TS	2.92 ± 0.12 ^e^	4.13 ± 0.35 ^b^
	SPS	3.32 ± 0.44 ^de^	5.61 ± 0.52 ^a^
	POS	6.00 ± 0.53 ^a^	5.75 ± 0.23 ^a^
HMMA	ISP	- ^(3)^	1.16 ± 0.31 ^C^
	CS	-	1.51 ± 0.49 ^BC^
	PS	-	2.63 ± 0.58 ^AB^
	TS	-	1.50 ± 0.16 ^BC^
	SPS	-	1.90 ± 0.54 ^ABC^
	POS	-	2.78 ± 0.35 ^A^

^(1)^ Low- and high-moisture meat analogs are abbreviated as LMMA and HMMA, respectively. ^(2)^ ISP: Isolated soy protein; CS: corn starch; PS: pea starch; TS: tapioca starch; SPS: sweet potato starch; POS: potato starch. ^(3)^ Expansion ratio was determined only for low-moisture meat analogs (LMMA), as expansion is suppressed under high-moisture extrusion conditions due to the use of a cooling die. ^(4)^ Values with different lowercase letters in the same column indicate significant differences within LMMA, and values with different uppercase letters in the same column indicate significant differences within HMMA (*p* < 0.05), as determined by Tukey HSD test.

**Table 2:** Textural properties and cutting strength of extruded meat analogs with different starches.

Starch Types		Springiness (%)	Chewiness (g)	Cohesiveness (%)	Cutting Strength (g/cm^2^)	
					Vertical Direction	Parallel Direction
LMMA ^(1)^	ISP ^(2)^	92.28± 1.13 ^ab(3)^	951.07 ± 101.48 ^ab^	82.04 ± 4.25 ^a^	372.59 ± 82.20 ^a^	256.03 ± 56.84 ^a^
	CS	89.98 ± 2.82 ^ab^	378.21 ± 110.05 ^c^	74.53 ± 7.54 ^ab^	136.23 ± 23.31 ^b^	96.149 ± 28.96 ^b^
	PS	89.48 ± 3.46 ^abc^	523.41 ± 164.52 ^c^	67.12 ± 22.91 ^ab^	226.39 ± 37.56 ^b^	153.27 ± 10.90 ^b^
	TS	87.27 ± 2.12 ^bc^	1229.59 ± 209.60 ^a^	65.46 ± 10.20 ^ab^	369.98 ± 69.28 ^a^	151.61 ± 16.61 ^b^
	SPS	84.60 ± 3.59 ^c^	1016.97 ± 206.47 ^ab^	57.34 ± 10.70 ^b^	220.60 ± 26.61 ^b^	124.19 ± 32.28 ^b^
	POS	92.64 ± 1.22 ^a^	869.20 ± 112.24 ^b^	84.38 ± 4.89 ^a^	224.69 ± 37.94 ^b^	114.48 ± 21.88 ^b^
HMMA	ISP	84.06 ± 1.37 ^A^	2570.18 ± 177.26 ^A^	50.75 ± 0.57 ^A^	3379.32 ± 526.22 ^A^	2477.09 ± 341.49 ^A^
	CS	81.77 ± 2.00 ^A^	1619.78 ± 203.26 ^B^	45.80 ± 1.13 ^BC^	2292.61 ± 94.13 ^B^	1936.60 ± 285.52 ^BC^
	PS	73.58 ± 2.44 ^B^	1425.49 ± 159.33 ^B^	43.77 ± 1.36 ^BC^	2816.83 ± 246.89 ^AB^	2161.63 ± 456.81 ^AB^
	TS	67.66 ± 0.04 ^C^	446.71 ± 234.43 ^C^	41.82 ± 0.03 ^C^	12,491.29 ± 2319.29 ^B^	7787.57 ± 1304.05 ^CD^
	SPS	63.87 ± 0.07 ^C^	369.29 ± 117.49 ^C^	37.16 ± 0.03 ^D^	1246.25 ± 182.31 ^C^	8717.24 ± 2992.75 ^D^
	POS	76.18 ± 0.03 ^B^	1045.65 ± 331.85 ^B^	46.82 ± 0.01 ^AB^	2440.24 ± 321.43 ^B^	1456.665 ± 127.21 ^CD^

^(1)^ Low- and high-moisture meat analogs are abbreviated as LMMA and HMMA, respectively. ^(2)^ ISP: Isolated soy protein; CS: corn starch; PS: Pea starch; TS: tapioca starch; SPS: sweet potato starch; POS: potato starch. ^(3)^ Values with different lowercase letters in the same column indicate significant differences within LMMA, and values with different uppercase letters in the same column indicate significant differences within HMMA (*p* < 0.05), as determined by Tukey HSD test.

**Table 3:** Effects of starch type on texturization degree and structural integrity of extruded meat analogs.

	Starch Types	Degree of Texturization	Integrity Index (%)
LMMA ^(1)^	ISP ^(2)^	1.46 ± 0.14 ^c(3)^	91.21 ± 0.76 ^a^
	CS	1.47 ± 0.20 ^c^	91.19 ± 1.30 ^a^
	PS	1.47 ± 0.15 ^c^	93.53 ± 1.78 ^a^
	TS	2.42 ± 0.22 ^a^	88.42 ± 1.31 ^a^
	SPS	1.86 ± 0.36 ^bc^	93.93 ± 1.03 ^a^
	POS	1.98 ± 0.10 ^b^	94.42 ± 3.34 ^a^
HMMA	ISP	1.47 ± 0.41 ^A^	18.65 ± 6.51 ^B^
	CS	1.21 ± 0.11 ^A^	44.63 ± 4.84 ^A^
	PS	1.35 ± 0.19 ^A^	44.86 ± 5.36 ^A^
	TS	1.66 ± 0.43 ^A^	21.75 ± 4.60 ^B^
	SPS	1.38 ± 0.28 ^A^	5.15 ± 3.15 ^B^
	POS	1.48 ± 0.09 ^A^	40.91 ± 6.51 ^A^

^(1)^ Low- and high-moisture meat analogs are abbreviated as LMMA and HMMA, respectively. ^(2)^ ISP: Isolated soy protein; CS: corn starch; PS: pea starch; TS: tapioca starch; SPS: sweet potato starch; POS: potato starch. ^(3)^ Values with different lowercase letters in the same column indicate significant differences within LMMA, and values with different uppercase letters in the same column indicate significant differences within HMMA (*p* < 0.05), as determined by Tukey HSD test.

## Data Availability

The original contributions presented in this study are included in the article. Further inquiries can be directed to the corresponding author.
